# Surface Energy of Filtration Media Influencing the Filtration Performance against Solid Particles, Oily Aerosol, and Bacterial Aerosol

**DOI:** 10.3390/polym11060935

**Published:** 2019-05-29

**Authors:** Seojin Jung, Jaejin An, Hyungjin Na, Jooyoun Kim

**Affiliations:** 1 Department of Textiles, Merchandising and Fashion Design, Seoul National University, Seoul 08826, Korea; wjdwls04079@snu.ac.kr; 2 Medical Convergence Textile Center, Gyeongbuk Technopark, Gyeongsangbuk-do 38412, Korea; anjaejin@gbtp.or.kr (J.A.); hjna@gbtp.or.kr (H.N.); 3 Research Institute of Human Ecology, Seoul National University, Seoul 08826, Korea

**Keywords:** filtration, electrospun web, surface energy, electret, particle, aerosol, bacteria

## Abstract

Particulate airborne pollutants are a big concern to public health, and it brings growing attention about effective filtration devices. Especially, particulate matters smaller than 2.5 µm can reach the thoracic region and the blood stream, and the associated health risk can be exacerbated when pathogenic microbials are present in the air. This study aims at understanding the surface characteristics of nonwoven media that influence filtration performance against solid particles (sodium chloride, NaCl), oily aerosol (dioctyl phthalate, DOP), and *Staphylococcus aureus* (*S. aureus*) bacteria. Nonwoven media of polystyrene (PS) fibers were fabricated by electrospinning and its pristine surface energy (38.5 mN/m) was modified to decrease (12.3 mN/m) by the plasma enhanced chemical vapor deposition (PECVD) of octafluorocyclobutane (C_4_F_8_) or to increase (68.5 mN/m) by the oxygen (O_2_) plasma treatment. For NaCl particles and *S. aureus* aerosol, PS electrospun web showed higher quality factor than polypropylene (PP) meltblown electret that is readily available for commercial products. The O_2_ plasma treatment of PS media significantly deteriorated the filtration efficiency, presumably due to the quick dissipation of static charges by the O_2_ plasma treatment. The C_4_F_8_ treated, fluorinated PS media resisted quick wetting of DOP, and its filtration efficiency for DOP and *S. aureus* remained similar while its efficiency for NaCl decreased. The findings of this study will impact on determining relevant surface treatments for effective particulate filtration. As this study examined the instantaneous performance within 1–2 min of particulate exposure, and the further study with the extended exposure is suggested.

## 1. Introduction

The deteriorating air quality and the associated health impact is a big concern worldwide, and this raises the importance of respiratory protection against the airborne pollutants. Ambient pollutants are comprised of fine dust, liquid mist, metal fume, volatile organic compounds (VOCs), and biological matters such as allergens and microbials [1]. Of special concern is particulate matter smaller than 2.5 µm (PM 2.5) [2] as they can reach the thoracic region and the blood stream, and can cause serious health risks including respiratory and cardiovascular diseases [3]. The associated risk can be exacerbated when pathogenic microbials including bacteria and virus are present in the air [4,5]. Coughing, sneezing, and talking can generate microbial aerosols in the air, potentially carrying infectious diseases. The role of air purifying devices becomes more important in the occurrence of viral pandemics such as SARS, H1N1 influenza, and the secondary bacterial infections associated with viral pandemics [1,4,5,6].

Airborne particles and aerosol can be captured by a single fiber mainly by the mechanisms of interception, inertial impaction, diffusion, and gravitational settling [7,8,9]. When a fiber is electrostatically charged, electrostatic attraction and induced polarization additionally contribute to the capture of particles, either charged or uncharged, thus the filtration efficiency can be improved [7,10,11,12,13]. Thus, commercially available filters are mostly electrically charged (electret) by the various charging processes. As particles are captured by contacting on a single fiber, not by sieving, the fibrous structure with high specific area and with long air pathways are usually beneficial for filtration [14]. Often, a high filtration efficiency is traded off by a high pressure drop, and the respirator users are forced to compromise the protection performance with the breathing comfort [15]. Thus, the filter development has been driven to give superior filtration efficiency at the low pressure drop. Most commercial filter media used for respirators are made of nonwovens manufactured by meltblown (MB) process, due to its openness of structure and tortuous air pathway, which allows breathability while capturing particles.

As electrospinning process has been used as a versatile method of fabricating submicron to micron fibers with various morphological structures [16,17,18,19,20], electrospun webs have been studied for its applicability as filter media [9,15,21,22,23,24,25,26,27,28,29,30,31,32,33,34]. Electrospun nanofibers can be relevant for filter application due to its high specific surface area and the inherent initial charges resulting from the electric field-driven process. However, nanofibers with high packing densities may result in a high pressure drop that leads to earlier clogging [15,32], thus, design parameters including fiber size, solidity, thickness, and basis weight should be prudently chosen to satisfy both filtration efficiency and pressure drop [15,22,23,29,32,34]. To account for both efficiency and pressure drop, the concept of quality factor (QF) is commonly employed [15] where a higher QF indicates a higher efficiency at the same level of pressure drop. The QF can be used as an inherent quality of a filter media, that is, multi-layers of filter media may have a higher filtration efficiency and a higher pressure drop than a single layer, but this multi-layer will have the same QF as the single layer of the same kind filter media. 

Certifications of filtering performance are different depending on countries and regions, and the corresponding test methods are different. In general, filtering respirators are tested against solid particles such as NaCl and oily aerosol such as paraffin oil or dioctyl phthalate (DOP). However, the details of test methods such as air velocity and particle size are different depending on the methods. Standard methods in Europe (CEN) and Australia and New Zealand (AS/NZ) evaluate the instantaneous filtration results, while US (National Institute for Occupational Safety and Health, NIOSH) and China (GB) refer to the loading results [35]. Regardless of differences, the applied challenging agents are commonly NaCl and oily aerosol (DOP or paraffin oil). 

While bacterial filtration is of significant interest for many parties, it has been rarely studied in the field of air filtration [36,37,38]. Bacterial filtration has been studied mostly for liquid filters, due to an urgent impact of bacterial presence in water on public health [36,39,40]. Silver nanoparticles have been frequently employed as a biocide in the liquid filter material [39,40], but the potential long-term impact on human health has not been fully understood yet. Previous studies [37,41] reported about the applicability of soy-protein fibers to bacterial filtration, and reported that the positive charges of amino acid enhanced the adhesion of aerosolized bacteria onto the protein nanofibers [11,12,37]. In other studies, the surface wettability was reported to influence on the bacterial adhesion [20,42,43,44]. Though the wetting criteria advantageous for bacterial adhesion are not conclusive, a moderately hydrophobic surface generally showed a higher level of bacterial adhesion [20]. Most commercially available air filters are made from hydrophobic materials such as polypropylene (PP), but the filtration efficiency against bacteria on this surface has been rarely examined. Furthermore, the effect of surface wettability or surface energy on filtration performance against various particulate matters has not been investigated yet. It is generally assumed that the bacteria filtering mechanism follows the same particle capture mechanism, and if this is true, the filtration efficiency for bacteria would be positively correlated with the result with other particles. 

**Figure 1:**
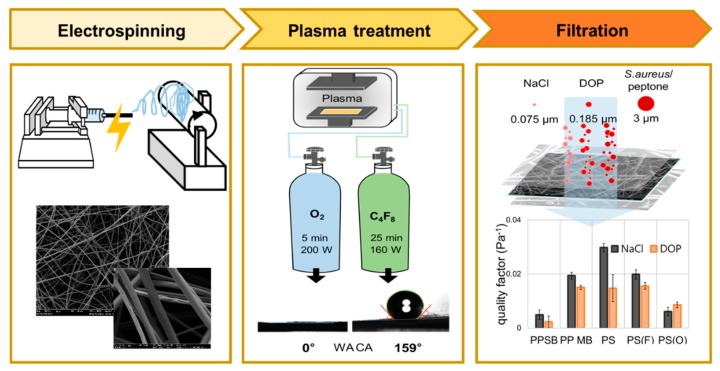
Schematic overview of study.

This study aims at understanding the effect of surface wettability, or surface energy of nonwoven media, on filtration of different types of challenging agents including solid particles of NaCl, oily aerosol of DOP, and *Staphylococcus aureus (S. aureus)* bacteria. As filter media, polystyrene (PS) electrospun web (ES), PP meltblown (MB) electret, and PP spunbond (SB) web were compared. The surface property of PS ES was modified to decrease by the plasma enhanced chemical vapor deposition (PECVD) using C_4_F_8_ [45,46,47], and to increase by the O_2_ plasma treatment [16,20,48]. The surface chemistry, surface energy and the wettability were examined for the different webs, and the filtration performance was discussed in association with those properties. As the filtration efficiency is generally inversely correlated with the pressure drop, the QF accounting for both factors was used as an inherent quality of filter media. A schematic overview of this study is presented in Figure 1. The ultimate goal of this study is to understand whether filtering performance of bacterial aerosol is different from that of other particles/aerosol, and to provide a design insight for filter media that would be desirable for filtration of various matters. The findings of this study will impact on determining relevant surface treatments for effective air filtration.

## 2. Materials and Methods 

### 2.1. Materials

Polystyrene (PS) pellets (Mw~350,000) was purchased from Sigma Aldrich (St. Louis, MO, USA). *N*,*N*-dimethylformamide (DMF), and tetrahydrofuran (THF) were obtained from Fisher Scientific (Hampton, NH, USA). Polypropylene (PP) spunbond web (SB) was obtained from Dainnuri (Incheon, Korea), and PP meltblown (MB) electret filter was obtained from Korea Institute of Industrial Technology (KITECH) (Cheonan, Korea). PP film was made by casting PP resin obtained from SK Global Chemical Co., Ltd. (Seoul, Korea). Polystyrene films were obtained from Goodfellow (Huntingdon, UK). Sodium chloride (ACS grade) was purchased from Showa Chemical Industry Co., Ltd. (Meguro-ku, Tokyo, Japan) and dioctyl phthalate (DOP, ACS grade) was obtained from Junsei Chemical Co., Ltd. (Chuo-ku, Tokyo, Japan). Diiodemethane (99.0%) was obtained from Alfa Aesar (Haverhill, MA, USA). Peptone water was made by adding 20 g of Luria-Bertani broth (LB broth, 0.5% NaCl with pH 7.5) to 1 L distilled water and autoclaving it for 15 min at 120 °C prior to use. LB broth was purchased from Daihan Scientific (Wonju, Korea). Octafluorocyclobutane (C_4_F_8_) gas and oxygen gas (O_2_) were purchased from Union Gas (Yongin, Korea).

### 2.2. Fabrication of Filter Media

The PS resin was dissolved in a solvent mixture of DMF and THF in a 1:3 *v/v* ratio to make a 20% (*w/v*) pre-spinning solution. An electrospinning apparatus (ESR200D, NanoNC, Seoul, Korea) was set to feed the PS pre-spinning solution at 6 mL/h and 12 kV of voltage. The needle gauge of 23 (inner diameter, 0.34 mm) was used as the electrospinning tip, and the tip to collector distance was set 14 cm. The electrospun fibers were collected on a drum collector rotating at 100 rpm. The chamber temperature and relative humidity (RH) were 15 ± 5 °C and 30 ± 5% RH, respectively. The produced electrospun web had the basis weight of about 14 g/m^2^ and thickness of 0.13 mm. Solidity and the porosity of the web were calculated by Equations (1) and (2), respectively, to compare the open porosity of different webs. 

The surface chemistry of electrospun web was modified to vary surface energy of materials. To increase the surface energy, the PS web was subject to O_2_ plasma treatment for 5 min at 200 W at 160 sccm in a Covance^TM^ plasma system (FemtoScience, Hwaseong, Korea). To lower the surface energy, the material was treated for C_4_F_8_ PECVD for 25 min at 200 W with 100 sccm. The generated frequency of plasma was 50 kHz in both O_2_ and C_4_F_8_ PECVD treatment.

Solidity = m/[A⋅t⋅ρ](1)

Porosity (%) = [1 − solidity] × 100 (%)(2)

- m (g): sample mass
- A (${\mathrm{cm}}^{2}$): sample area
- t (cm): sample thickness
- ρ (g/${\mathrm{cm}}^{3}$): polymer density (1.04 g/cm^3^ for PS, 0.95 g/cm^3^ for PP)

### 2.3. Characterization

The atomic concentrations (%) of various surfaces were analyzed using an X-ray photoelectron spectrometer (XPS, Axis Supra^TM^, Kratos Analytical, Manchester, UK). The surface morphology of web was observed using a field-emission scanning electron microscope (FE-SEM, Supra 55VP, Carl Zeiss, Jena, Germany), with prior Pt coating at 30 mA for 200 s using a sputter coater (EM ACE200, Leica, Wetzlar, Germany). Fiber diameter of the web was measured from SEM images using ImageJ software (v.1.52e, NIH, USA), randomly selecting 30 fibers from the images.

Pore size distribution of web was measured using the capillary flow porometer (CFP 1500AE, PMI Inc., Ithaca, NY, USA). Static contact angle (CA) and shedding angle (ShA) were measured using an optical tensiometer (Theta Lite, KSV Instruments Ltd., Espoo, Finland). The CA of a 3.4 µL of liquid drop was measured after 2–5 s upon deposition. For shedding angle (ShA), the onset of tilting angle where a 12.5 µL liquid drop rolls at least 2 cm was recorded. For CA and ShA, five measurements from different surface locations were conducted.

The surface energies of untreated and treated PS were calculated using Equations (3)–(6) by measuring the contact angles (CAs) of water and diiodomethane (DM) on flat film surfaces, adopting the Owens-Wendt model [49]. For surface energy estimation, only flat film surfaces were measured for CAs to meet the assumption of the model. The surface energies of the PS webs were regarded as the same as those of PS films.

(3) $$\gamma_{\mathrm{SL}}{=\;\gamma }_{S}{+\;\gamma }_{L}-\;2\sqrt{\gamma_{S}^{d}\cdot \gamma_{L}^{d\;}}-\;2\sqrt{\gamma_{S}^{p}\cdot \gamma_{L}^{p}}$$

(4) $$\gamma_{S}{=\;\gamma }_{\mathrm{SL}}{+\;\gamma }_{L\;}\;\cos\theta$$

(5) $$\gamma_{L}(1+\cos\theta )=\;2\sqrt{\gamma_{S}^{d}\cdot \gamma_{L}^{d}}\;-\;2\sqrt{\gamma_{S}^{p}\cdot \gamma_{L}^{p}}$$

(6) $$\gamma_{S}{=\;\gamma }_{S}^{d}{\;+\;\gamma }_{S}^{p}$$

- θ: contact angle of liquid on solid surface
- $\gamma_{\mathrm{SL}}$: interfacial energy between solid and liquid
- $\gamma_{S}$: surface energy of solid
- $\gamma_{S}^{d}$: dispersive component surface energy of solid
- $\gamma_{S}^{p}$: polar component surface energy of solid
- $\gamma_{L}$: surface energy of liquid
- $\gamma_{L}^{d}$: dispersive component surface energy of liquid
- $\gamma_{L}^{p}$: polar component surface energy of liquid

### 2.4. Filter Performance

For the filter test, a single layer PS ES or PP MB was layered with PP SB’s at the top and the bottom each, to prevent from tearing of PS ES or PP MB during the test. Automatic Filter Tester (TSI 8130, TSI Inc., Shoreview, MN, USA) was used to evaluate the instantaneous pressure drop and the filtration efficiency (or % penetration) during 1 min of exposure to NaCl or DOP. As particulate matters, NaCl (count median diameter ~0.075 µm) and DOP (count median diameter ~0.185 µm) were used as representative solid particles and oily aerosol, respectively. The filter area of 40 cm^2^ was exposed to the air flow of either NaCl (~18.6 g/L) or DOP (~67.8 g/L) at 28.3 L/min, and this corresponds to 11.8 cm/s of face velocity. The % penetration of NaCl and DOP passing through the filter was determined by the relative particle concentration upstream and downstream of the test sample. The quality factor (QF), the relative filtration efficiency to the pressure drop, was calculated as Equation (7) to compare the filtration performance of media with different basis weight and thickness.

(7) $$\mathrm{Quality}\;\mathrm{factor}\;(\mathrm{Pa}{-}^{1})\;=\;\ln\left(\frac{\%\;\mathrm{penetration}/100\%}{\mathrm{pressure}\;\mathrm{drop}\;(\mathrm{Pa})}\right)$$

### 2.5. Bacterial Filtration

**Figure 2:**
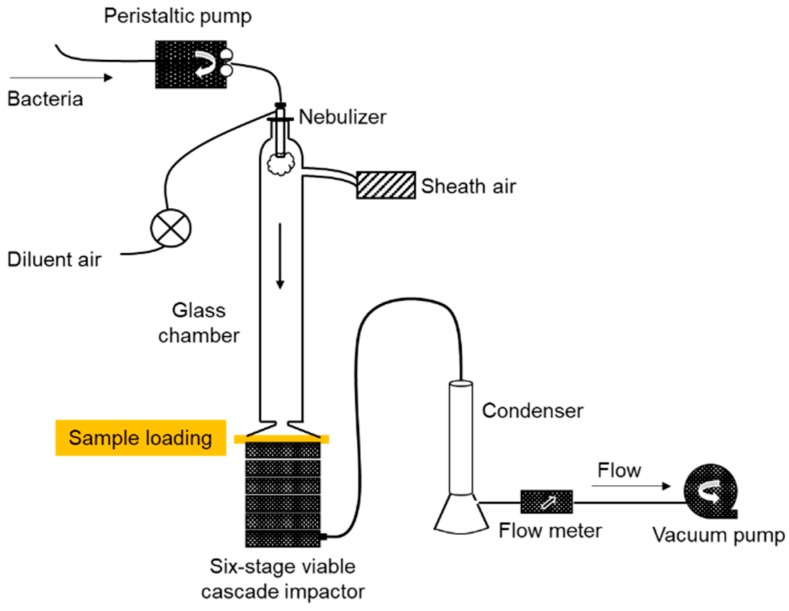
Illustration of bacterial filtration test.

The bacterial filtration efficiency, or bacterial penetration of filter media was evaluated according to ASTM F2101-14 using a custom-designed equipment, as illustrated in Figure 2. *Staphylococcus aureus* (6538, ATCC, Manassas, VA, USA) was cultured in the sterilized tryptic soy broth (211825, BD, Sparks, MD, USA) for 24 h and diluted with sterilized peptone water. The diluted bacteria were delivered by the peristaltic pump, then nebulized at (2200 ± 500 viable particles and mean size of 3.0 ± 0.3 µm) for 1 min per test. The filter media was clamped between a six-stage viable cascade impactor (TE-10-800, TISCH Environmental, Cleves, OH, USA) and a glass chamber, then the aerosol was pulled through the filter sample at 28.3 L/min for 2 min, using a vacuum attached to the viable cascade impactor. The viable particles by each size range of cascades were collected on a tryptic soy agar (236950, BD, USA) placed in a six-stage cascade impactor. Then positive control tests were performed without the clamped sample. The tryptic soy agar plates were incubated at 37 °C for 48 h, then the positive hole numbers were counted in each plate. The counted numbers were converted to the colony forming unit (CFU) of *S. aureus*, using the converting factor provided in the manufacturer’s reference for the viable cascade impactor. Likewise, a positive control was run as the same procedure without sample loading. The bacterial penetration (%) was determined by the relative CFU penetrated through the filter sample to the positive control, as in Equation (8).

Bacterial penetration (%) = (S/P) × 100 (%)(8)

- P: CFU converted from the positive hole numbers for the positive control
- S: CFU converted from the positive hole numbers when filter sample was loaded

## 3. Results

### 3.1. Electrospun Filter Media with Various Surface Energies

**Figure 3:**
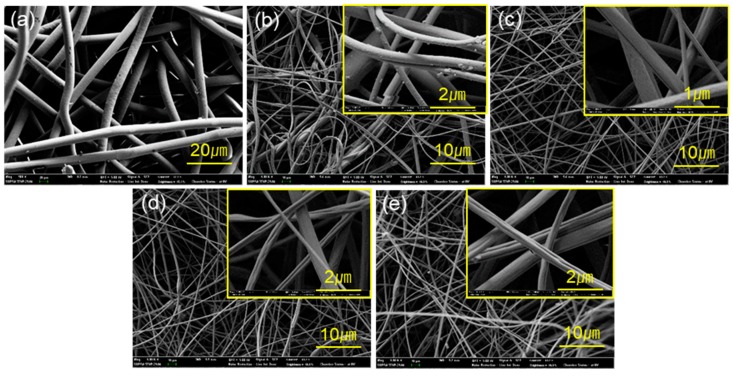
Scanning electron microscope (SEM) images of different webs. (**a**) polypropylene (PP) spunbound (SB); (**b**) PP meltblown (MB); (**c**) polystyrene (PS) electrospun web (ES); (**d**) PS(F) ES; (**e**) PS(O) ES (O_2_ plasma-treated web).

The filter media used for particulate respirators are often electrostatically charged in a separate step to improve the relative filtration efficiency to the pressure drop (dP). In electrospinning, the produced fiber can have the inherent static charges as the fiber is formed in an electric field [15], and it does not necessarily require an additional step of the charging process. If a polymer with the low dielectric constant is used for electrospinning, the electric field created by static charges would remain rather longer [10]. The dielectric constants (unitless) of PP and PS are about 2.2–2.4 and 2.4–2.7, respectively, and can be good candidates for electret filters. As polystyrene (PS) can be electrospun easily with various options of solvents, PS was used for electrospinning to fabricate electret filter media. Furthermore, small fibers could develop a high pressure drop, the electrospinning condition was adjusted to form relatively thick fibers with mean diameter of about 2–3 μm (Figure 3). 

According to the US NIOSH standard [50], filters are classified as N, R, and P types, depending on the filtration efficiency against NaCl test particles (N-type) and DOP test aerosol (R and P-types). The oily aerosol in general deteriorates the filtration efficiency of electret filters rather quickly, because the oil drops spreads on fiber surface, masking the charged sites. Thus, if the surface can be treated to reduce the wettability against oily liquid, then the performance may last longer. With such assumptions, the wettability or the surface energy of PS electrospun web (ES) was modified using the plasma process, in order to investigate the resulting filtration performance. The surface energies of the PS webs were modified to decrease by the C_4_F_8_ PECVD treatment, and to increase by the O_2_ plasma treatment.

The surface chemistry of different polymer surfaces was examined by the atomic concentration (%) via XPS analysis. The XPS analysis was not possible for the nonwoven webs as the small fibers from the web had movement during the x-ray analysis. Instead of nonwoven media, the untreated and plasma-treated PS and PP flat films were subject to XPS analysis. From the XPS results (Table 1), untreated PS and PP films consisted of hydrocarbons only; O_2_ plasma-treated PS film exhibited about 15% of O and 85% of C and C_4_F_8_ PECVD-treated film had about 29% of F and 71% of C. The results confirmed that the plasma treatment effectively modified the surface chemistry of substrates. 

The oxygen plasma usually removes organic contaminants from the surface and leaves a free radical on the surface, increasing the surface energy [51]. After O_2_ plasma treatment of PS electrospun web (ES), the weight of the PS(O) ES (O_2_ plasma-treated web) was reduced, probably due to the removal of surface hydrocarbons to some extent [52]. After C_4_F_8_ PECVD, the weight and thickness of the fluorinated web, PS(F) ES, remained as similar to those of untreated PS ES. The characteristics of nonwoven media used in this study are shown in Table 2. From Figure 3, the morphologies of webs were not changed much after the plasma treatments of O_2_ and C_4_F_8_; while the weight and the thickness of PS(O) ES were reduced, there was no observable damage shown for PS(O) ES fibers.

The surface energy of substrates was estimated by measuring CAs of water (WA) and diiodomethane (DM) using the Owens–Wendt model [16,49,53] (Table 3). The surface energy of PS was significantly changed with plasma treatments. With the C_4_F_8_ PECVD process, the surface energy was changed from 38.5 mN/m (untreated PS) to 12.3 mN/m, and with O_2_ plasma, it was greatly increased to 68.5 mN/m. The O_2_ treated surface increased the polar component significantly, while the C_4_F_8_ PECVD surface lowered the total surface energy. PP had a low total surface energy with the near zero polarity. The result confirmed that the modification of surface chemistry effectively changed the surface energy. 

As a result, the wettability of web was greatly affected. The wettability of different web surfaces was compared in Table 4. The water (WA) CA of PP SB, PP MB, PS ES, and PS(F) ES were all as high as >154°. As PS ES itself is very hydrophobic, WA CA could not discriminate the wettability of PS ES and PS(F) ES. However, the ShA from PS(F) ES (11°) was much lowered than that from the PS ES (41°), showing that the water adhesion on the PS(F) ES was considerably reduced. The ShA measurement is often a better discriminating parameter of wettability when the surface is very hydrophobic [53]. When the wettability was increased by the O_2_ plasma process, the PS(O) ES was wet immediately upon the deposition of water drop. The CAs of different liquid including DOP and peptone water (PEP) were measured on surfaces, to examine the wettability against the test aerosols of DOP and bacterial media. After C_4_F_8_ PECVD, the wettability of DOP was considerably reduced, to its CA of 145°. PEP is a bacterial broth medium used for cultivation of bacteria, which is comprised of peptic digest of animal tissue and sodium chloride, and its CA on surfaces was lower in general than WA CA. 

### 3.2. Filtration Performance against NaCl and DOP

**Figure 4:**
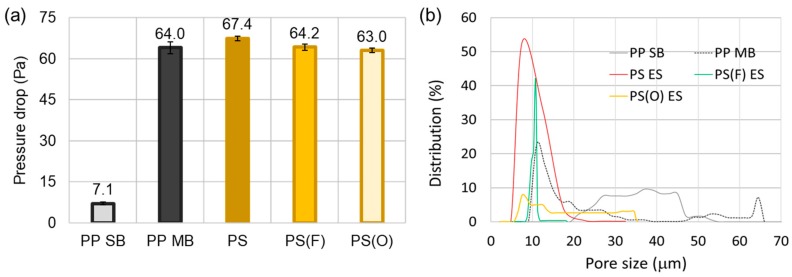
Pressure drop (**a**) and pore size distribution (**b**) of nonwoven media.

The instantaneous filtration performance of different webs was compared for NaCl solid particles and DOP oily aerosol. PP SB is hardly used as an effective filter media. In this study, two-layer PP SB was used as top and bottom layers to give mechanical durability to electrospun web (ES) and meltblown web (MB) during the test. A two-layer PP SB web was tested as a control sample. From Figure 4a, the pressure drop (dP) of two-layer PP SB was as low as ~7 Pa, and its contribution to pressure drop would be negligible when layered with PP MB and PS ES. PS ES showed slightly higher pressure drop (67.4 Pa) than PP MB (64.0 Pa). In general, the web with a larger fiber size has a larger pore size. Thus, the web with larger fibers is anticipated to give a lower pressure, if other parameters are the same. The pore size distribution for each web is shown in Figure 4b. As all tested webs had 89%–90% porosity, a slightly higher pressure drop of PS ES may be attributed to the smaller pore sizes than PP MB. A lower pressure drop of PS(O) ES, compared to PS ES, corresponds to the lowered basis weight after O_2_ plasma treatment, which was due to the removal of hydrocarbons in slight amounts, resulting in creation of large pores (Figure 4b). However, the pressure drop difference among the samples was not much except for PP SB.

**Figure 5:**
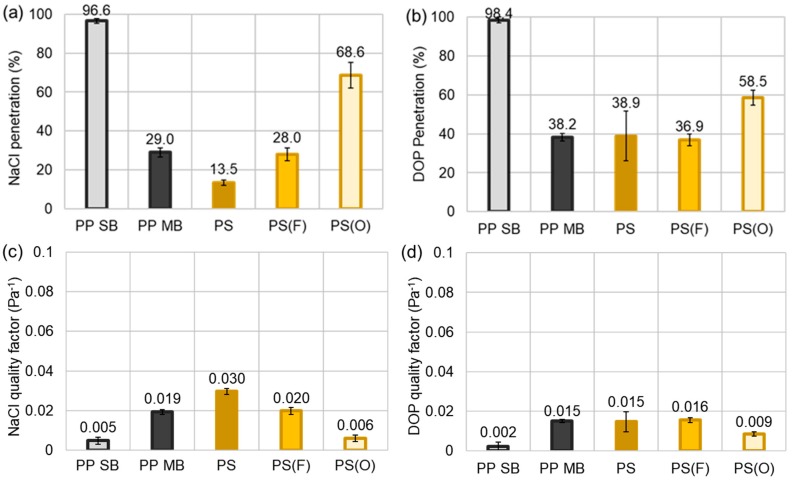
Filtration performance for NaCl and DOP. (**a**) % Penetration of NaCl; (**b**) % Penetration of DOP; (**c**) Quality factor for NaCl; (**d**) Quality factor for DOP. Note: PP SB is two layers of PP SB.

From Figure 5, the level of penetration for DOP appeared much higher than that for NaCl. As anticipated, PP SB had negligible filtration efficiency for both NaCl and DOP. PS ES (13.5%) showed lower penetration than PP MB (29.0%) for NaCl particles, while it had the similar level of DOP penetration as the PP MB. The higher efficiency may be attributed to the static charges of electrospun web and smaller fiber sizes. When PS ES was subject to plasma treatment, the NaCl penetration significantly increased both for C_4_F_8_ and O_2_ treatments. Particularly, PS(O) ES, which was hydrophilized, lost the most of filtration function. As the porosity and the pressure drop of the plasma-treated web remained similar to that of untreated PS ES, the loss of filtration efficiency of PS(O) ES is probably due to the loss of static charges after activation with O_2_ plasma. From the XPS analysis, PS(O) film surface has about 30% of oxygen group, which may lead to the better electrical conductivity in the environment. The dissipation of static charges and the decrease of electric field of electrospun web would result in the lowered filtration efficiency for both NaCl and DOP. 

While the effect of C_4_F_8_ on filtration is not very clear, the static charges on PS(F) ES seemed to be partially lost, adversely affecting the NaCl filtration. It was inferred that the coating with fluorinated compound on PS web partially masked the surface charges, deteriorating the charging effect to some extent. Though it was speculated that the lowered DOP wettability of PS(F) ES would be advantageous for DOP filtration, the result did not show the significant effect, because of the compromised effect between the masked charges and the lowered DOP wettability.

For the filter media that is used for particulate respirator, low penetration and low pressure drop are preferable as they can be translated as high protection and breathing comfort for users. As penetration and pressure drop often trade off each other, the quality factor (QF) is calculated to account for both penetration and pressure drop factors simultaneously (Equation (7)). The QF rather determines the inherent quality of the filter media, and a higher QF is translated to have a higher efficiency at the same pressure drop. In Figure 5c–d, QFs at the instantaneous exposure of NaCl and DOP are compared. In general, PS ES showed the highest QF for NaCl, functioning as an effective filter media that can be applied to an N-type class filter. The QFs against DOP were about the same for PP MB, PS ES, and PS(F) ES. To design a high performing final product with a low pressure drop, it is important to use a filter media with the high QF. For instance, to make a filter construction with 95% efficiency against NaCl particles at the face velocity of ~11.8 cm/s, two layers of PS ES (efficiency ~98.3%; pressure drop ~135 Pa) or three layers of PP MB (efficiency ~97.6%, pressure drop ~192 Pa) would be needed. In this case, using the PS ES is beneficial because it uses less material, and it gives higher protection at a lower pressure drop. Likewise, once a filter media with the high QF is obtained, the layer construction can be designed to meet the specific performance requirement.

### 3.3. Bacterial Filtration Performance

**Figure 6:**
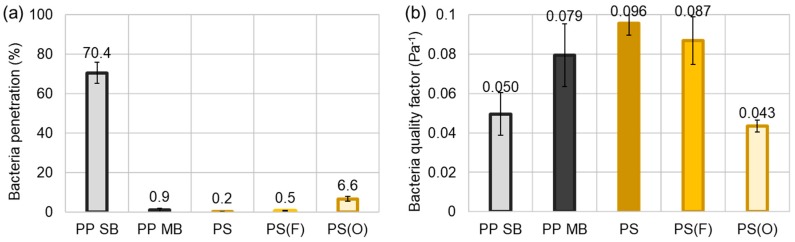
*S. aureus* bacteria filtration. (**a**) % Penetration; (**b**) Quality factor. Note: PP SB is two layers of PP SB.

Figure 6 shows the bacterial filtration performance against *S. aureus*, the gram-positive bacteria with spherical shape. The bacteria aerosolized in peptone media was used for the test. Bacterial penetration was all low for PS ES, PS(F) ES, and PP MB webs. The lowered wettability against the bacterial broth, peptone water, was not specifically beneficial for bacterial filtration. Compared to PP MB, PS ES, and PS(F) ES showed slightly higher QF against *S. aureus* bacteria. The higher QF of PS webs may be attributed to the smaller fiber sizes that allowed better mechanical capture of *S. aureus* aerosol (mean diameter of ~3 μm). Like NaCl and DOP, filtration performance against bacteria was deteriorated for PS(O) ES. As the pressure drop and porosity of PS(O) ES were not considerably different from the untreated, this lowered efficiency is mainly because of the loss of charges by the O_2_ plasma, not because of the structural changes. Bombardment with active oxygen groups during the plasma process affected the static charges, and the hydrophilic surface of PS(O) ES would dissipate static charges more easily. Overall PS(O) ES showed the significantly deteriorated filtration performance for all three types of particulate substances, NaCl, DOP, and *S. aureus*. Though the surface charge influenced the bacterial filtration efficiency, the mechanical capture of bacterial aerosol seemed to be an important mechanism in this case, because the mean diameter of *S. aureus* aerosol was about 3 μm, which is much larger than NaCl particles and DOP aerosol. Surface charges would be most beneficial for capturing most penetrating particle size of about 0.3 μm. On the other hand, for large particles and aerosol such as the *S. aureus* aerosol in this study, interception would be a main capture mechanism, where compact structure becomes advantageous for particle capture. A two-layer PP SB, hardly functioning as filter media, also displayed about 30% of efficiency (70% penetration) against S. aureus bacteria. 

It should be noted that the results here show the instantaneous penetration within 1–2 min of exposure: 1 min for NaCl and DOP and 2 min for *S. aureus*. With the continued loading of particles and aerosol, the influence of fiber layers and thickness would be important on depth loading of particulate matters [22]. Further study with the extended exposure to challenging agents is needed to simulate the actual use of filter products. 

## 4. Conclusions

This study examined the influence of surface energy of filter media on the filtration performance against solid particles (NaCl), oily aerosol (DOP), and *S. aureus* bacteria. As a versatile process of manufacturing electret filter media, electrospinning was employed to fabricate a fibrous assembly having tortuous air pathways. The PS electrospun web (ES) had fibers in the mean diameter ≥ 2 μm. The porosity of all the webs tested in this study was very similar (~90%), but the pressure drop ranged differently depending mostly on fiber sizes. The surface energy of PS ES (38.5 mN/m) was modified to decrease (12.3 mN/m) by the plasma enhanced chemical vapor deposition (PECVD) of C_4_F_8_ or to increase (68.5 mN/m) by the O_2_ plasma treatment. As a result, the wettability of the treated web was greatly affected. Particularly, the wettability against DOP after C_4_F_8_ PECVD treatment was considerably reduced to its CA of 145°. While it was speculated that the lowered wettability against oily liquid may be advantageous for DOP filtration, the result did not show the significant effect of lowered wettability on performance. PS(F) ES showed the lowered filtration performance against NaCl particles than PS ES. It was inferred that the coating with the fluorinated compound on the electret fibers partially masked the surface charges, deteriorating the surface charges to somewhat extent. For NaCl and *S. aureus*, PS ES showed higher quality factor than PP MB electret that is readily available for commercial products. The O_2_ plasma treatment of PS media significantly deteriorated the filtration efficiency with minimal change of pressure drop, due to the quick dissipation of static charges after O_2_ plasma treatment. Bacterial filtration efficiency was all high for PS ES, PS(F) ES, and PP MB webs. The lowered wettability against the bacterial broth, peptone water, was not specifically beneficial for bacterial filtration. Compared to PP MB, PS ES and PS(F) ES showed slightly higher QF against *S. aureus* bacteria. Like NaCl and DOP, O_2_ plasma treatment considerably deteriorated filtration performance against *S. aureus*. The findings of this study will impact on determining the relevant surface treatment to design effective particulate filtration. This study examined the instantaneous performance within 1–2 min of particulate exposure. As the filter media should function for the continued loading of particulate substances, further study with the extended exposure is suggested to simulate the actual use of filter media.

## Figures and Tables

**Table 1:** X-ray photoelectron spectrometer (XPS) atomic concentration (%) of surfaces.

Surface	C (%)	O (%)	F (%)
PP	100	-	-
PS	100	-	-
PS(F)	71	-	29
PS(O)	85	15	-

Note: The XPS analysis was conducted on the film surfaces that were treated or untreated. The fibrous surface was not measurable by XPS due to the presence of protruding fine fibers.

**Table 2:** Characteristics of webs.

Web	Basis Weight (g/m^2^)	Thickness (mm)	Fiber Diameter (μm)
PP SB	23 (± 1)	0.23 (± 0.04)	23.0 (± 1.5)
PP MB	30 (± 2)	0.32 (± 0.07)	4.1 (± 1.5)
PS ES	14 (± 1)	0.13 (± 0.03)	2.2 (± 1.1)
PS(F) ES	13 (± 3)	0.13 (± 0.02)	2.5 (± 1.3)
PS(O) ES	10 (± 1)	0.10 (± 0.03)	3.1 (± 1.5)

**Table 3:** Surface energy of different surfaces.

Sample	Contact Angle (°)		Surface Energy (mN/m)		
	WA	DM	γ	γ^d^	γ^p^
PP	105	63	26.9	26.7	0.16
PS	110	45	38.5	38.0	0.46
PS(F)	120	91	12.3	12.2	0.07
PS(O)	21	55	68.5	25.6	42.9

Note: **γ**, total surface energy; **γ^d^** and **γ^p^**, dispersive and polar components of surface energy, respectively. For WA, the dispersive and polar components of 21.8 mN/m and 51.0 mN/m, respectively, were used [54]. For DM, the dispersive and polar components of 50.4 mN/m and 0.4 mN/m, respectively, were used [54].

**Table 4:** Surface wettability of different webs.

Measure	PP SB	PP MB	PS ES	PS(F) ES	PS(O) ES
WA CA	154° (± 2.7)	154° (± 1.8)	157° (± 1.5)	159° (± 4.0)	0°
WA ShA	34° (± 1.7)	14° (± 1.1)	41° (± 0.5)	11° (± 2.0)	NA
DOP CA	0°	0°	0°	151° (± 2.5)	0°
PEP CA	121° (± 1.2)	144° (± 2.8)	145° (± 1.8)	153° (± 2.5)	0°

Note: WA, water; CA, contact angle; ShA, shedding angle; PEP, peptone water.
