# Erratum: Surface Energy of Filtration Media Influencing the Filtration Performance against Solid Particles, Oily Aerosol, and Bacterial Aerosol. *Polymers* 2019, *11*, 935

**DOI:** 10.3390/polym12051189

**Published:** 2020-05-22

**Authors:** Seojin Jung, Jaejin An, Hyungjin Na, Jooyoun Kim

**Affiliations:** 1 Department of Textiles, Merchandising and Fashion Design, Seoul National University, Seoul 08826, Korea; wjdwls04079@snu.ac.kr; 2 Medical Convergence Textile Center, Gyeongbuk Technopark, Gyeongsangbuk-do 38412, Korea; anjaejin@gbtp.or.kr (J.A.); hjna@gbtp.or.kr (H.N.); 3 Research Institute of Human Ecology, Seoul National University, Seoul 08826, Korea

The authors wish to make a change to the published paper [1]. In the original manuscript, there are mistakes in the Equations (5) and (7). The corrected Equations (5) and (7) are presented below.

(5) $$\gamma_{L}(1+\cos\theta )=2\sqrt{\gamma_{S}^{d}\cdot \gamma_{L}^{d}}\;+2\sqrt{\gamma_{S}^{p}\cdot \gamma_{L}^{p}}$$

θ: contact angle of liquid on solid surface

$\gamma_{L}$: surface energy of liquid

$\gamma_{S}^{d}$: dispersive component surface energy of solid

$\gamma_{L}^{d}$: dispersive component surface energy of liquid

$\gamma_{S}^{p}$: polar component surface energy of solid

$\gamma_{L}^{p}$: polar component surface energy of liquid

(7) $$\mathrm{Quality}\;\mathrm{factor}\;({\mathrm{Pa}}^{-1})=\frac{-\ln(\frac{\%\;\mathrm{penetration}}{100\;\%}\;)}{\mathrm{pressure}\;\mathrm{drop}\;(\mathrm{Pa})}$$

The authors apologize for any inconvenience caused and the change does not affect the scientific results. The manuscript will be updated, and the original will remain online on the article webpage at https://www.mdpi.com/2073-4360/11/6/935.
